# Visual Analysis of the Daily QA Results of Photon and Electron Beams of a Trilogy Linac over a Five-year Period

**DOI:** 10.4236/ijmpcero.2015.44035

**Published:** 2015-11-09

**Authors:** Maria F. Chan, Qiongge Li, Xiaoli Tang, Xiang Li, Jingdong Li, Grace Tang, Margie A. Hunt, Joseph O. Deasy

**Affiliations:** 1 Dept. of Medical Physics, Memorial Sloan-Kettering Cancer Center, New York, NY; 2 Dept. of Physics, The City University of New York, New York, NY

**Keywords:** Linac QA, Data Visualization, Daily Output, Radiotherapy

## Abstract

Data visualization technique was applied to analyze the daily QA results of photon and electron beams. Special attention was paid to any trend the beams might display. A Varian Trilogy Linac equipped with dual photon energies and five electron energies was commissioned in early 2010. Daily Linac QA tests including the output constancy, beam flatness and symmetry (radial and transverse directions) were performed with an ionization chamber array device (QA BeamChecker Plus, Standard Imaging). The data of five years were collected and analyzed. For each energy, the measured data were exported and processed for visual trending using an in-house Matlab program. These daily data were cross-correlated with the monthly QA and annual QA results, as well as the preventive maintenance records. Majority of the output were within 1% of variation, with a consistent positive/upward drift for all seven energies (~+0.25% per month). The baseline of daily device is reset annually right after the TG-51 calibration. This results in a sudden drop of the output. On the other hand, the large amount of data using the same baseline exhibits a sinusoidal behavior (cycle = 12 months; amplitude = 0.8%, 0.5% for photons, electrons, respectively) on symmetry and flatness when normalization of baselines is accounted for. The well known phenomenon of new Linac output drift was clearly displayed. This output drift was a result of the air leakage of the over-pressurized sealed monitor chambers for the specific vendor. Data visualization is a new trend in the era of big data in radiation oncology research. It allows the data to be displayed visually and therefore more intuitive. Based on the visual display from the past, the physicist might predict the trend of the Linac and take actions proactively. It also makes comparisons, alerts failures, and potentially identifies causalities.

## 1. Introduction

Pre-treatment daily dosimetry monitoring including x-ray and electron output constancy together with the functional safety checks is recommended for all medical accelerators [1–3]. The daily tests include parameters that can affect dose to the patient, such as dosimetric output constancy, geometric accuracy of lasers, optical distance indicator, and field size, as well as other safety tests. These daily tests are typically carried out by the morning warm-up radiation therapist using a cross-calibrated dosimetry system. Commercial flat-panel multidetector arrays with appropriate buildup material may be also used for daily quality assurance (QA). The absolute dose output and other dosimetry tests are also performed in the monthly QA test with higher-level equipment at tighter tolerance criteria. This provides confidence in the daily device and will identify trends that may otherwise go undetected over the course of a long period of time.

In addition to keeping parameters within specification, it is important for a physicist to monitor trends in the machine performance. A well-designed trending tool may help to predict future problems and allow proper planning of action level [4]. Large longitudinal data visualization analysis differs from traditional statistical analysis because it is dynamic due to its data capacity and content, and more advanced processing methods have made it possible to analyze the dynamic visualization [5]. The objective of this study is to utilize data visualization to trend the behavior of photon and electron beams of a new linear accelerator (Linac) in our radiotherapy center. This work devotes to discovering the common behaviors of the Linac output, so that physicists can be more confident in predicting machine’s future behavior and taking action in a planned way before the tolerance level is reached. Preventive adjustment is more efficient than post-event action (adjusting after the tolerance level is reached); on the other hand, this intuitive adjustment grounding on everyday observations can be more economical than the more frequent prescribed preventive maintenance.

## 2. Materials and Methods

**Figure 1:**
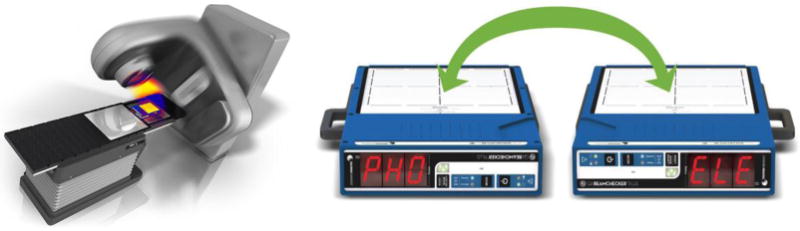
(a) QABC+ on a Linac couch to measure daily outputs for all 7 energies before treating cancer patients; (b) QABC+ device: flips between photons and electrons measurement.

**Figure 2:**
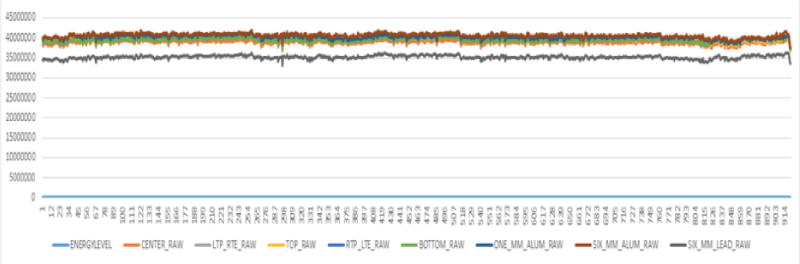
Unfiltered raw data extracted from QABC+ for 5-year period.

**Figure 3:**
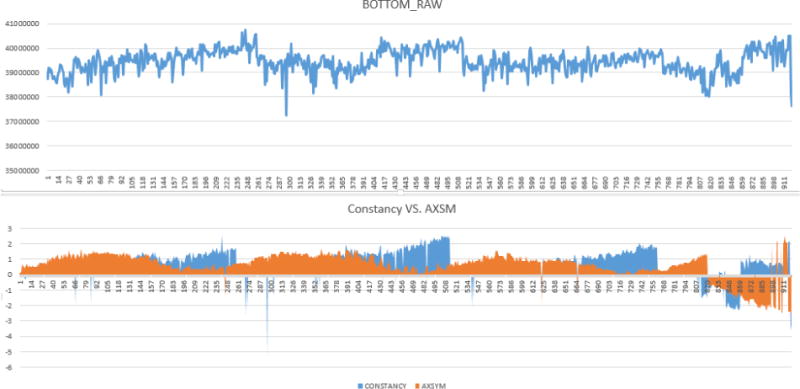
(a) 6 MV raw data extracted from QABC+; (b) Combined 6MV output constancy and axial symmetry along the timeline on the X-axis. The Y-axis represent of the percentage difference from baseline.

A Trilogy Linac (Varian Medical Systems, Palo Alto, CA) equipped with dual photon energies (6 and 15 MV) and five electron energies (6, 9, 12, 16, and 20 MeV) was commissioned in early 2010. Daily Linac QA tests including the output constancy, flatness, and beam symmetry (for both radial and transverse directions) were measured with an ionization chamber array device, QA BeamChecker Plus (QABC+) (Standard Imaging Inc., Wisconsin, US), for the past five years (3/16/2010 – 3/02/2015). There are 8 vented ion-chambers in the device: 1 center detector, 4 quadrant detectors (7.5 cm from center), and 3 energy identification chambers. Figure 1(a) and Figure 1(b) show the setup of QABC+ on a Linac couch for QA tests and the device flips on different side for photon/electron measurement. This task is performed by a radiation therapist on a daily basis to ensure the Linac output within tolerances in compliance with the AAPM TG142 recommendation. All data are stored in the database of the QABC+ software. Figure 2 demonstrates a set of typical unfiltered raw data extracted from the daily device. Figure 3(a) shows the 6 MV raw data extracted from QABC+; and Figure 3(b) plots the 6 MV output and axial symmetry (CONSTANCY vs. AXSYM) along the timeline over 5-year period. CONSTANCY is in percentage of the output variation to the baseline; AXSYM is beam symmetry along the axial direction; TRSYM is symmetry along the transverse direction; and FLATNESS is uniformity over the radiation field.

After the measurement data were exported for each of the seven energies, they were then processed for visual trending using an in-house program developed with Matlab (v8.1.0.604). Output constancy data were plotted for all energies. For both photon energies, the symmetry plots were fitted with a third-order sinusoidal function and the flatness plot was fitted with sum of sine with a 4^th^ order function (the curve fitting tool provided by Matlab). These daily measurement data were cross-correlated with monthly QA and annual QA data, as well as the preventive maintenance records.

In addition, a multiple regression analysis was performed by creating the scatter plot matrix that provides a quick detection of the correlation between variables. The scatter plot matrix for all seven energies were created by the commercial statistical software Minitab 17 (Windows). Each variable pair is the same-day measurements. Linear regression model was fitted in each of these plots. The correlation coefficients were calculated by using Matlab statistical function corrplot().

## 3. Results

**Figure 4:**
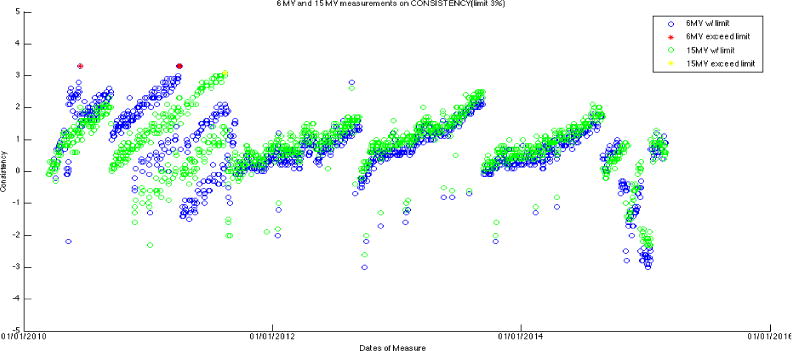
Constancy of two photon energies in 5-year timeline.

**Figure 5:**
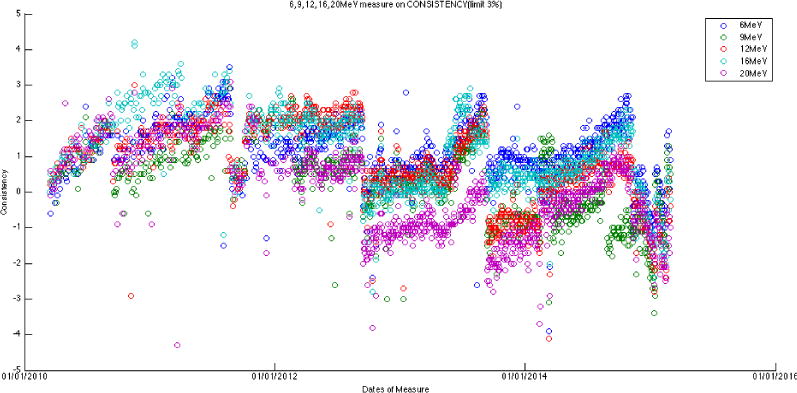
Constancy of five electron energies in 5-year timeline.

The output of the Linac is mostly within 1% of variation, with a consistent positive drift for all seven energies (~+0.25% per month). The output is adjusted and corrected every year in September, when annual TG-51 calibration is performed. This results in the sudden drops of output back to the baseline in every September in the data trend. Figure 4 and Figure 5 are the constancy plots for photons and electrons over the 5-year period, respectively.

**Figure 6:**
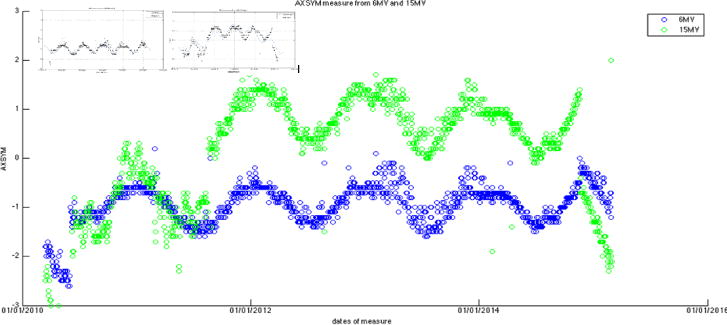
Filtered photon axial symmetry data with curve fitting functions in the 2 separate plots.

**Figure 7:**
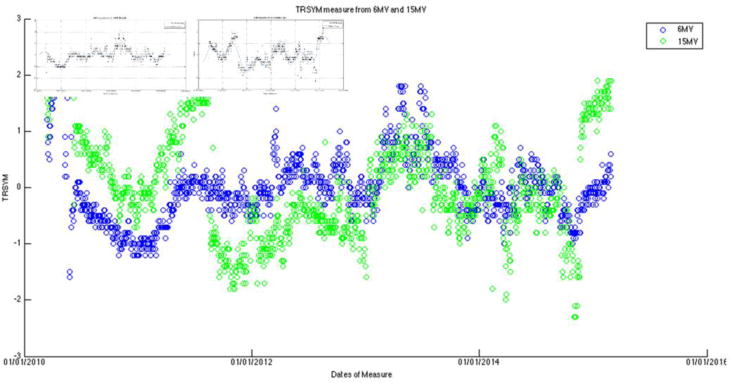
Filtered photon transverse symmetry data with curve fitting functions in the 2 separate plots.

**Figure 8:**
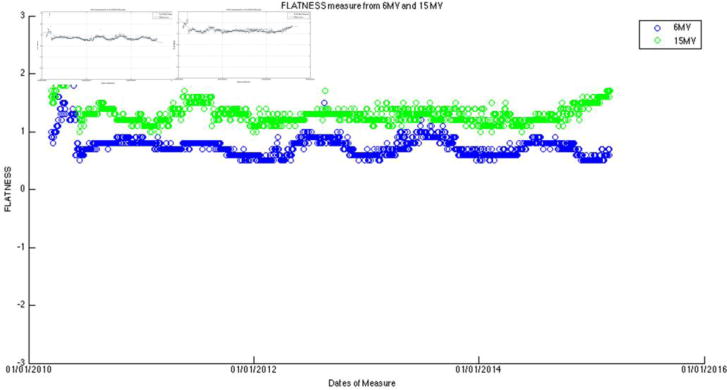
Filtered photon flatness data with curve fitting functions in the 2 separate plots.

**Figure 9:**
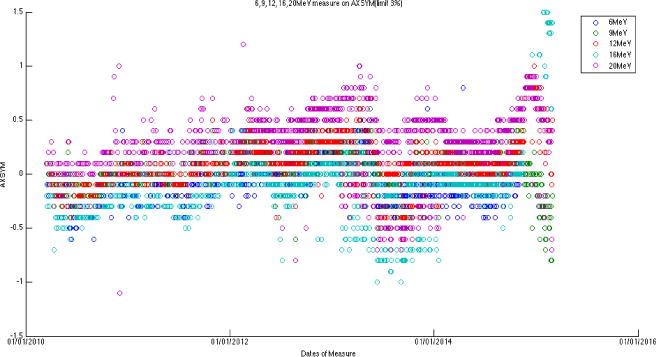
Axial symmetry for five electron energies.

**Figure 10:**
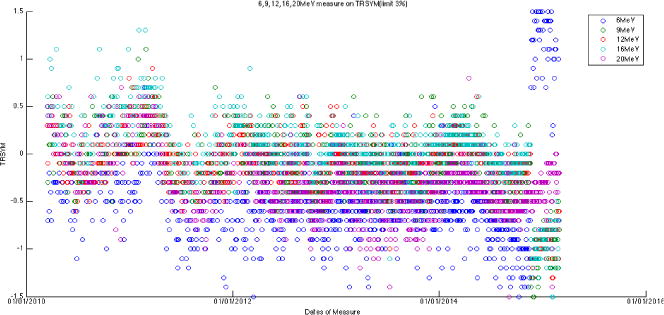
Transverse symmetry for five electron energies.

**Figure 11:**
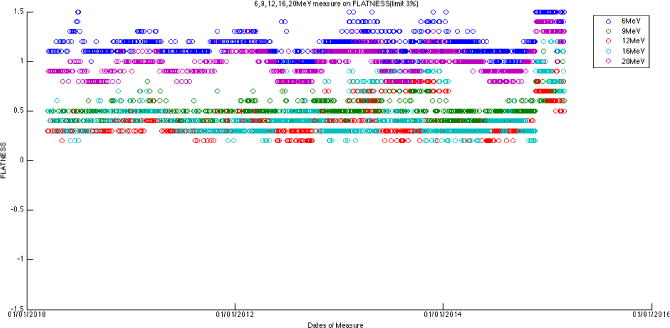
Flatness (data combined both axial and transverse) for five electron energies.

The large amount of data using the same baseline exhibits a sinusoidal behavior (cycle = 12 months; amplitude = 0.8%, 0.5% for photons, electrons, respectively) on symmetry and flatness when normalization of baselines is accounted for. Figures 6–11 are the AXSYM, TRSYM, and FLATNESS plots for photons and electrons over the 5-year period, respectively. The photon plots were fitted with a sinusoidal function (small windows). We did not show the fitting function for the electron plots for better clarity.

**Figure 12:**
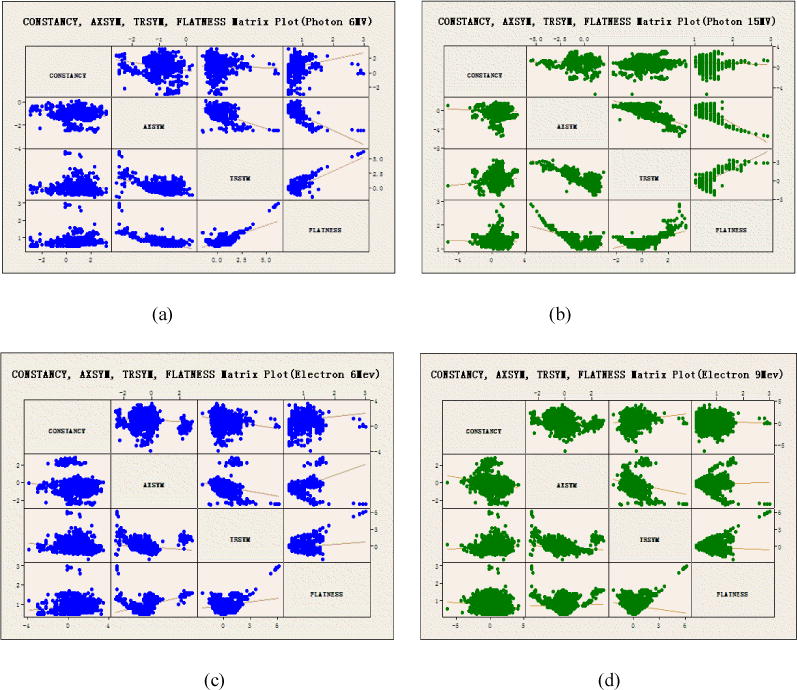

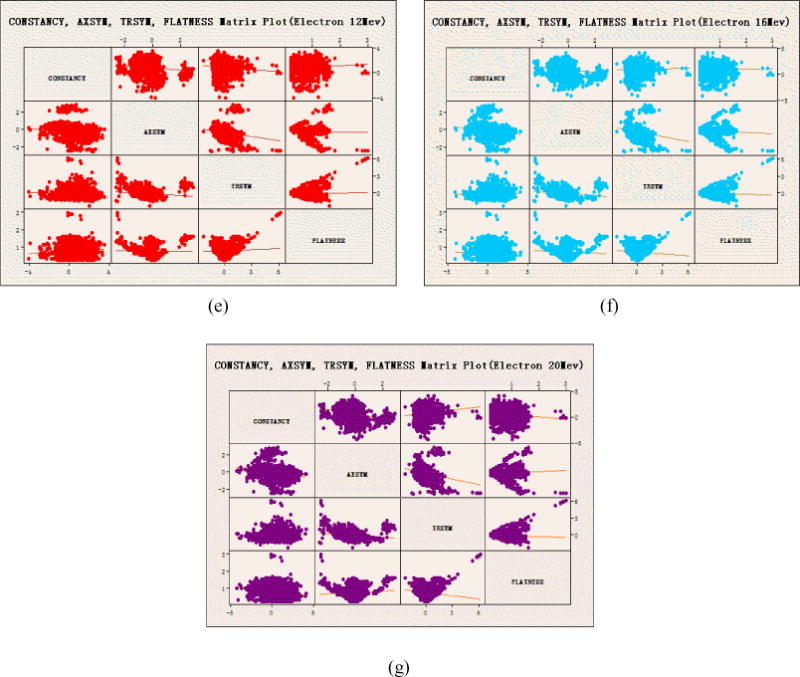
(a)–(g) Scatter-plot matrices of the four variables for all seven energies.

A scatter plot matrix helps knowing one dosimetric parameter’s behavior, which could assist predicting others. Figures 12(a)–(g) show the scatter plot matrix for all seven energies. The scatter plot matrix for photons (both 6 MV and 15 MV) shows relatively strong negative correlation between AXSYM and FLATNESS (correlation coefficient (CC): −0.67 and −0.71), negative correlations between AXSYM and TRSYM (CC: −0.51 and −0.76), and positive correlations between TRSYM and FLATNESS (CC: 0.67 and 0.62). However, for different electron energies, this correlation relationship is not consistent: AXSYM and TRSYM show strong positive correlation (CC: 0.78) in 6 MeV electron plot, while negative correlation (CC: −0.52) in 16 MeV electron plot, and no correlation in other energies (−0.25 ~ 0.29). AXSYM and FLATNESS show positive correlation (CC: 0.62) only in 6 MeV electron plot and almost no apparent correlation in other energies (CC: −0.4 ~ 0.26). TRSYM and FLATNESS show positive correlation in 6 MeV energy electron plot (CC: 0.52) but negative correlations in 9, 12, and 16 MeV electrons (CC: −0.52, −0.57, −0.59), and no correlation (CC: −0.12) in 20 MeV plot. Over all seven energies, CONSTANCY is not correlated to any of the other three variables, while there are correlations among the symmetry and flatness for both photon energies. For electrons, only 6 MeV shows correlations between symmetry and flatness, similar to photons. The least correlation among variables is 20 MeV. The order from strong to week correlations in electrons is 6 MeV, 16 MeV, 9 MeV, 12 MeV, and 20 MeV, respectively. The knowledge gained from this study would help the physicist better understand the different behavior or character for each beam.

## 4. Discussion

The output of the Linac is mostly within 1% of variation, with a consistent positive drift for all seven energies (~+0.25% per month), which is closely consistent with Grattan and Hounsell’s report [6] on “average monthly increase of 0.26+/−0.009% over the course of the first 4 years of use”. However, in their study, they reported that the output increase then reduces to −0.03 +/−0.002% for the following three years. For this aspect, we will continue to monitor the trend and see if this would be in the same direction as theirs. The phenomenon of output drift for new Linacs is due to air leakage of the over-pressurized sealed monitor chambers made by the specific vendor [6]. Furthermore, our data is very consistent with the seasonal fluctuation (quasi-periodic behavior) found in the previous studies [6] and [7]. Particularly, the sinusoidal behavior is in agreement with the observations in seasonal variation described by other researchers [6]–[8]. This is probably due to the incoming power to the circuit boards, which can be affected by the environmental factors year-round, and the varying output that could also cause more and less scatter from the electronics and structures surrounding the chambers in the QABC+. The environmental factors include the fixed timing of the startup of the air conditioning and heating in our building.

Grattan and Hounsell [6] reported their analysis of Varian linear accelerators output trends up to 7 years. The results indicated that the response was generally consistent within each set of accelerators with different monitor chamber designs. The chambers used in their 2100C/D Linac resulted in an increase in measured output over time for the first 4 years of use, which is nearly the same model as our Trilogy Linac. Similar to our findings, their output response trend appeared the same (upward shift) for all clinical energies used on the 2100C/D Linac - 6, 15 MV x-ray beams, and 4, 6, 9, 12, 16 and 20 MeV electron beams. By tracking these changes it has been possible to predict the response over time to allow appropriate adjustments and gain confidence in machine performance. It has also provided data to indicate the need for planned preventative intervention and indicate if the monitor chamber response is behaving as expected. In our case, when visualizing our output and symmetry plots, we realized some unusual behavior (non-trending) starting from the end of 2014. By investigating further, we found out that the memory of the QABC+ was beginning to fail since that time. Then we sent out the unit to the manufacturer for repair including upgrading the firmware and software.

Bartolac and Letourneau [7] characterized underlying seasonal variations in Linac output over 3 years for 15 Linacs, in particular 6 MV photon beams. Runtime plots of output revealed sinusoidal, seasonal variations that were consistent across all units, irrespective of manufacturer, model or age of machine. The average amplitude of the variation was on the order of 1%. The peak and valley occurred in early April and September, respectively. Coincidentally our symmetric plots for all energies were observed almost the same pattern (cycle = 12 months; amplitude = 0.8%, 0.5% for photons, electrons, respectively). Characterization of cyclical seasonal trends allows for better separation of potentially innate accelerator behavior from other behaviors (e.g. linear trends), which could indicate service requirements.

Hossain [8] also reported that for 3 Linacs with 9 beams, output can increase by about 2–4% per year over a period of more than three years, if artificially removing the adjustments by physicists that were done once every 3–6 months. Similar to our setting, the output was measured using devices with ion chambers. Similar to our findings (Figures 6–10), they found quasi-periodic behavior manifested in the seasonally averaged output, showing annual variability with negative variations in the winter and positive in the summer. This trend was weakened when the daily output was normalized by the monthly-calibrated output, indicating that the variation of the periodic component may be intrinsic to both the Linacs and the daily measurement devices. To our understanding, the stability of some Linac components can vary with seasonal temperature changes, incoming power, and main circuit board, which affects the beam symmetry.

Uddin [9] studied short and long term drifts in outputs of Linacs by fitting empirical mathematical models to the QA measurements. They found that long-term drifts were well modeled (≤ 1.5%) by either a straight line or a single-exponential function. A drift of 2% occurred in 12–18 months. The shortest drift times of only 2–3 months were observed for some new accelerators just after the commissioning, but they stabilized during the first 2–3 years. The short-term reproducibility and the long-term stability of local constancy checks, carried out with a sealed plane parallel ion chamber, were also estimated by fitting empirical models to the QA measurements. The reproducibility was 0.3–0.6% depending on the positioning practice of a device. Long-term instabilities of about 0.3%/month were observed for some checking devices. Luketina *et al* [10] reported that one Varian Linac (SN42) was relatively stable with the output generally drifting between +/− 1% and the other newer linear (SN1027) had a consistent increase in the average output of about 2.5% per year over a period of five years.

Overall, our output and symmetry trends are consistent with all reports in the literature for new Linacs using the sealed monitor chambers from the specific vendor. In this work, we used data visualization tools to efficiently evaluate the pattern of output fluctuation over a long period of time, and detect any unusual events in daily performance. Moreover, our analysis uncovers the correlation relationships between different variables, allowing the prediction of their behavior. This work shows a strong potential for applying data visualization tools to clinical medical physics field, in particular to improve the effectiveness of quality control program. Future works include developing new data visualization techniques, utilizing machine learning to predict the behaviors of variations in machine’s output, and further exploring the correlation relationship among different features.

## 5. Conclusions

Data visualization is a new trend in the era of big data in radiation oncology research. It enables the past descriptive information not only be made more intuitive and enhanced support for data analyzing, but also can significantly enhance the expression of the pattern. Effective visualization helps physicists to predict the response over time and allows appropriate adjustments to maintain the Linac within tolerances. Data visualization is also an effective tool to perform data comparisons, alert failures, and potentially identify causalities.
